# What makes oncology nurses and registered nurses motivated to work in oncology specialty: a qualitative interview study

**DOI:** 10.1186/s12912-024-02620-6

**Published:** 2025-04-14

**Authors:** Omar Qaladi, Frank Donnelly, Ellen Davies, Gillian Harvey

**Affiliations:** 1 College of Nursing, King Saudi University, Riyadh, Saudi Arabia; 2 Adelaide Nursing School, The University of Adelaide, Adelaide, Australia; 3 Faculty of Health and Medical Science, Adelaide Health Simulation, The University of Adelaide, Adelaide, Australia; 4 College of Nursing and Health Sciences, Flinders University, Adelaide, Australia

**Keywords:** Oncology Nursing, Nursing Shortage, Nursing Retention, Nursing Turnover, Nursing Work environment

## Abstract

**Background:**

Oncology nursing in Saudi Arabia faces unique challenges, including a shortage of local nurses, cultural and linguistic barriers, and insufficient support systems. Understanding the factors that motivate, and challenge oncology nurses is crucial to addressing these issues and improving retention.

**Objectives:**

This study aims to explore the motivations and challenges of oncology nurses and registered nurses in Saudi Arabia, focusing on both intrinsic and extrinsic factors influencing their work in the oncology specialty.

**Methods:**

A qualitative exploratory descriptive approach was employed, involving semi-structured interviews with 19 participants, including oncology registered nurses (ORNs) and registered nurses (RNs) from various specialties at King Saud University Medical City in Riyadh. The data were examined through thematic analysis to uncover patterns and insights related to the participants’ experiences.

**Results:**

The study identified four primary themes: advantages of working in oncology, psychological difficulties, structural barriers, and workplace conditions affecting job satisfaction. Nurses reported a sense of achievement and personal fulfillment as key motivators. However, they also faced significant psychological challenges, including emotional exhaustion and inadequate psychological support. Structural barriers such as lack of specialized training and career pathways, as well as dissatisfaction with the salary scale, were major deterrents. Communication barriers and high workloads further reduced job satisfaction.

**Conclusions:**

This study, the first of its kind in Saudi Arabia, highlights the complex interplay of factors influencing oncology nurses' experiences. Addressing these issues through targeted interventions, improved training programs, competitive salaries, and better psychological support can enhance job satisfaction and retention of oncology nurses in Saudi Arabia.

## Introduction

Cancer is a global health problem with a 30% increase in diagnoses from 14.1 million in 2012 to 19.3 million in 2019 worldwide [1, 2]. It is the primary cause of mortality on a worldwide level [1, 2]. The cancer prevalence in Saudi Arabia exhibited a notable rise of 14.3% throughout the period from 2018 to 2020, with a 25.4% increase in cancer-related deaths from 10,200 to 13,150 cases. This rise can be attributed to multiple factors, including an aging population, increased calorific intake, and lifestyle changes [1, 2].

The rise in cancer cases has placed a significant demand on healthcare services and professionals in Saudi Arabia, particularly in nursing [3–5]. In response, the Saudi government has initiated measures such as the Saudization policy and the ambitious ‘Saudi Vision 2030’ project to expand the local nursing workforce [6, 7]. These initiatives aim to reduce the shortage of Saudi nurses and enhance their presence across all fields of nursing, with a particular focus on specialized areas like oncology and palliative care nursing [6].

Oncology nursing in Saudi Arabia faces several challenges, including unfavourable attitudes towards providing care for terminally ill patients [8], and a notable lack of knowledge about palliative care and pain management [9]. Furthermore, the proportion of Saudi nurses in oncology nursing is approximately 9%, significantly lower than the national average of 36.5% for Saudi nurses in the overall nursing workforce [3, 5]. Since most oncology nurses in Saudi Arabia are expatriates they struggle with fluency in Arabic, limiting their ability to communicate effectively with patients and potentially affecting the quality of healthcare services [6, 10].

To understand the challenges that face oncology nursing, several studies have investigated the issues in different countries [11–13]. A previous systematic review revealed a positive connection between nursing staff shortages and dissatisfaction with the nursing job, burnout, and stress [11]. A quantitative study was conducted in Saudi Arabia to investigate these factors in the context of oncology nursing, revealing that nurses’ attitudes and knowledge significantly impacted their willingness to work in oncology, with those possessing more positive attitudes and better knowledge being more likely to work in the field [5]. However, no qualitative study has explored this issue in further detail.

Therefore, this study aims to provide an in-depth understanding of the factors influencing nurses’ decisions to work in oncology by examining various perspectives. The findings from this study will inform strategies to attract and retain nurses in the oncology specialty, addressing the specific challenges and opportunities within the Saudi Arabian healthcare context.

## Methods

### Study design and aims

To understand the barriers and enabling factors perceived by oncology registered nurses and registered nurses in other specialties, a descriptive qualitative study was conducted at a university-teaching hospital in Riyadh, Saudi Arabia.

### Setting and sample

Participants for the study were recruited from King Saud University Medical City (KSUMC) in Riyadh, Saudi Arabia. KSUMC is a tertiary care academic medical center with around 1,500 beds. The study included two distinct groups of nurses: those specializing in oncology (ORNs) and those registered nurse working in various other specialties (RNs). Inclusion criteria for the ORNs group included both Saudi and expatriate nurses in order to capture their differing perspectives on the experience of working in oncology nursing in Saudi Arabia. The inclusion criteria for the RN group required participants to be Saudi nationals with experience in caring for oncology patients. This criterion was essential to capture their insights on the barriers and facilitators to working as an oncology nurse in Saudi Arabia. The determination of sample size in qualitative research is guided by the study’s purpose, philosophical assumptions, and the need for comprehensive understanding and exploration of the phenomenon [14]. An inadequate sample size can compromise the depth and richness of the data, thus impacting the study’s quality and credibility [15]. Achieving thematic saturation is crucial, as it indicates that additional sampling no longer yields new information, but rather confirms previously collected data [16, 17]. In this study, interviews were conducted until a point where no new significant information emerged, suggesting sufficient depth of data. Therefore, thematic saturation was reached after conducting ten interviews with ORNs and nine interviews with RNs from other specialties.

### Data collection

Semi-structured interviews were conducted by the lead author (OQ) who is a male through an online Zoom platform at times convenient for the participants. Before conducting the study, the lead author (OQ) received formal training and gained experience in qualitative research methodologies, including conducting interview-based studies, to ensure the rigor and quality of the data collection process. There was no prior relationship between the lead author (OQ) and the participants to ensure impartiality and reduce potential bias during the interviews. All participants confirmed reading and acknowledging the participation information sheet, which detailed the author’s academic background, professional role, research aims and interest in oncology nursing and qualitative research. All interviews were recorded with the participant’s consent. The duration of each interview session ranged from 30 to 40 min. The interview questions used for both groups of participants are presented in Supplementary Table 1.

Given that most Saudi nurses have limited proficiency in English, potentially affecting their ability to articulate their thoughts during interviews, and conversely, most expatriate nurses have limited proficiency in Arabic, the interviews were conducted in English for non-Arabic speakers and in Arabic for Arabic speakers. To ensure accuracy and preservation of meaning, the researcher followed Van Nes et al.’s guidelines for translating qualitative research data. No participants withdrew during the data collection phase [18].

The audio recordings of the interviews with Saudi and expatriate nurses were transcribed and translated into both Arabic and English. After transcription, each participant received a copy of their interview to verify the accuracy of the transcription. Since the co-authors did not speak Arabic, the Arabic transcriptions were translated into English prior to data analysis. This translation process was carried out by a bilingual scholar and the researcher, who is fluent in both languages, adhering to the guidelines suggested by Van Nes et al. [18]. The researcher met with the translator periodically to confirm the accuracy and meaning of the translations.

### Data analysis

Transcribed interviews were imported and analyzed using NVivo 12.6, following Braun and Clarke's six-step thematic analysis method [19, 20]. Each transcript was carefully reviewed while listening to the interview recordings to ensure accuracy. Initial codes were generated from noteworthy aspects in the dataset, which were then collated into potential themes. Themes were validated with participant quotes.

To ensure the study’s robustness, we adhered to Lincoln and Guba’s five criteria: credibility, dependability, confirmability, transferability, and authenticity [21, 22]. Participants verified their interview transcripts, and the researcher meticulously reviewed translations for accuracy. Regular research team meetings helped maintain objectivity, aligning findings with participants’ perspectives. Comprehensive details about the research approach, participants, and setting were provided to aid transferability. Authenticity was emphasized to vividly capture participants’ experiences.

## Results

### Participants’ demographic information

Nineteen interviews were carried out with two distinct groups of participants: ten Saudi and non-Saudi ORNs, as well as nine Saudi RNs from non-oncology clinical divisions. Tables 1 and 2 displays the demographic characteristics of the participants. All RN participants from the non-oncology hospital departments had previous expertise in providing care to patients with cancer. Encounters with oncology patients were typically brief, occurring during admission via the emergency department or in high-intensity environments like the critical care unit.

**Table 1:** The interview questions used with both groups of participants

1) How would you describe oncology nursing, what are the first things that come to your mind?
2) For ORNs participants
a) Ca n you tell me a bit about the ward or unit that you are working in now?
b) Did you specifically choose to work in oncology? And if so, why?
c) What are the aspects of working in oncology that you particularly enjoy?
d) Are there aspects of working in oncology that you find difficult or challenging?
3) For RNs participants
a) Ca n you tell me a bit about the specialty that you are working in now?
b) Ca n you tell me what experiences you've had working in oncology?
c) Have you ever considered working in oncology? Can you tell me more about that?
For Both ORN and RN participants
4) Do they have any thoughts or insights as to why relatively few Saudi nurses choose to work in oncology?
5) Is there anything further you would like to a dd about the role of nurses in the oncology specialty?

**Table 2:** Characteristics of interview participants

Category	Interview participants (*n* = 19)	
**Profession**	ORN *n* = 10 (%)	Non-oncology RN *n* = 9 (%)
Gender		
Male	4 (40)	5 (56)
Female	6 (60)	4 (44)
Age *M*	30.9	27.9
Marital status		
Single	5 (50)	4 (45)
Married	5 (50)	5 (55)
Highest level of nursing education		
Bachelor degree	10 (100)	9 (100)
Nationality		
Saudi	7 (70)	9 (100)
Non-Saudi	3 (30)	0 (0)
Nursing experience (years) *M*	7.5	4.5
Oncology experience (years) *M*	4.6	0

*M* Mean

Four primary themes were identified: the advantages of working in the oncology specialty, psychological difficulties in oncology, structural barriers hindering oncology nursing, and finally, the workplace conditions decreasing job satisfaction. Additionally, 13 subthemes were identified, three for the first three major themes, and four for the last one as presented in Table 3.

**Table 3:** Summary of major themes and subthemes of the qualitative study results

Major Themes	Subthemes
1.Advantages of working in oncology	1.1 Sense of achievement and fulfilment 1.2 Feeling valued as a Saudi nurse 1.3 Development opportunities
2.Psychological difficulties in oncology	2.1 Perceptions about oncology 2.2 Emotional exhaustion 2.3 Inefficient psychological support
3.Structural barriers hinder oncology nursing	3.1 Education and training in oncology nursing were not available 3.2 lack of formal pathway to work in oncology 3.3 Salary scale issue
4.Workplace conditions reduce job satisfaction	4.1 Communication barrier issue 4.2 Team-related issues 4.3 Workload-related issues 4.4 Length of shift preference

### Advantages of working in oncology

This main theme expressed by ORN participants’ related to their positive impression of their experience working in oncology nursing. The interview data showed that participating oncology nurses were attracted to, and consequently remained in this specialty for various reasons, such as psychological benefits and potential career advancement. This major theme had three subthemes: sense of achievement and fulfilment, feeling valued as a Saudi nurse and development opportunities.

### Sense of achievement and fulfilment

ORN participants experienced a feeling of accomplishment from achieving positive patient outcomes through effective communication skills. For example, if a patient is in a negative psychological state, a nurse may support in improving the patient’s mood: *“My experience has been very excellent, especially if I feel at the end of the day that I have improved the patient’s psychological condition or convinced them to follow the treatment plan.”*

### Feeling valued as a Saudi nurse

The presence of a common culture and language among the Saudi nurses had favourable outcomes, fostering a sense of appreciation and worthiness among Saudi citizen nurses. In addition, the shortage of Saudi nurses specializing in oncology has engendered a sense of distinction and high demand among Saudi patients for these nurses: *“I feel that the patient relaxes psychologically if they sees a Saudi person around him. I consider it a wonderful thing. We consider our role supportive.”*

### Development opportunities

Most ORNs find the oncology specialty appealing since it is continually changing, which has a good effect on their learning and career growth:*“Even after years of exposure to patients with different diagnoses and cases, there is still a lot that I am not aware of, so there are still many things I need to discover … [it] provides me with something that I am very eager to learn.”* Several ORN participants recognized the benefit of advancement in their careers, as the oncology nursing field is relatively new. Saudi nurses’ scarcity increased their opportunities for career advancement and development.“*I can participate in developing the oncology department in my hometown hospital. I will have an opportunity to form good relationships with my colleagues there, and I will have a role in transferring the experience I have gained from this hospital……*”.

## Psychological difficulties in oncology

This main theme is focused on the negative emotional reactions of both ORNs and RNs to situations experienced in the oncology workplace. This theme was subdivided into three subthemes.

### Perceptions about oncology

An obvious difference was seen between the two cohorts of participants, ORNs and RNs, regarding their expression of complications and challenges encountered in the specialty of oncology. RNs often described their experience with oncology patients as challenging because they frequently encountered individuals in the terminal stage of the disease, which placed emotional and professional demands on them. As one nurse explained: *“We see patients with cancer in the ER sometimes … who usually come at the end stage, and their condition has deteriorated”* highlighting the difficulty of providing care in such critical and emotionally intense situations*.*

Additionally, it was noticed that several nurses considered cancer an untreatable disease that starts with chemotherapy, advances to a decline in the patient’s condition, and terminates with death:* “Patients get chemotherapy and get tired and hurt and die; this is the information we have about it”*.

Both participant groups expressed great anxiety over occupational hazards. The primary reason for this concern among the majority of participating RNs was a lack of understanding regarding oncology nursing as a whole and, more particularly, chemotherapy treatment: “*I was so scared. I just saw the IV chemotherapy medication, how the chemo bag covered. I walked out of the room … and the idea that patients’ relatives were not allowed to enter the room during chemo. The matter was a little frightening to me as an intern”*. A number of the ORNs also expressed their worries for their future health outcome as a result of handling cancer treatments such as chemotherapy and radiation: *“We are risking our lives, we are risking our fertility, and we are taking a chance that we might get a cancer at the end of our life”*. Furthermore, some nurses stated that they were concerned about the health effects of working near chemotherapy or radiation during pregnancy: *‘The female nurses, especially those planning to get pregnant, should stay away from chemo and radiation and move to clinic work… there is a fear of deformities in the baby’.*

### Emotional exhaustion

This subtheme highlights the emotional exhaustion experienced by ORN participants after experiencing a traumatic incident as a consequence of their therapeutic engagement with cancer patients. The closeness interactions with cancer patients increased the nurse’s emotional reaction, particularly in situations where the condition of the patient deteriorated: *“Imagine the patient in front of me for 40 days with his son or daughter or his wife … and when the time comes to sit with them at the same table and tell them that their son has been moved from the primary team to the palliative team. Can you imagine how they reacted? That certainly affects us”*. As a result of this form of close relationship, a sudden deterioration in a patient’s condition greatly affected nurses emotionally. Moreover, the ORNs feel sad when seeing patients suffering from nausea, pain, and vomiting as chemotherapy adverse effects of: “*The difficult part is having to see patients suffer … during chemotherapy where you can see the side effects … nausea, vomiting and pain; nobody likes the feeling of nausea and vomiting … it’s very hard to look at them … nurses are the ones who are caring, assessing and attending the patients during all their sufferings”*.

Most of the ORN participants believed that an oncology nurse needed a level of emotional resilience to stay working there: *“The emotional tension is very high there and requires a person with a strong heart to keep working”*. However, Some RNs expressed a negative attitude towards oncology work: *“I have the passion, but I do not have the courage because I tried dealing with relatives who have cancer, and it was painful, so I made the decision not to specialize in this field”.*

### Insufficient psychological support for staff

Many participants reported their dissatisfaction with the [lack of] available services to support mental health and wellbeing in the hospital. Also, certain participants in the ORN study indicated a desire for psychological assistance from nurses who specialize in the same field, as they would be more able to understand their emotions and provide debriefing support: “*I’m not aware of any oncology nursing association here in Saudi; if we had one, we could encourage everybody to come to do activities yearly or every six months. That would make life happier because we could debrief and talk about our challenges working in oncology”*.

## Structural barriers that hinder oncology nursing

The structural obstacles that impede the specialization of oncology nursing, as perceived by RN and ORN participants, are outlined in this main theme, and three subthemes were found.

### Education and training in oncology nursing were not available

The majority of the RNs and ORNs participants stated that they did not obtain any oncology nursing education or training throughout their undergraduate nursing education: “*To be honest with you, throughout my five-year bachelor’s degree, I don’t remember studying oncology*”. The RN participants pointed out the same problem, and several RNs indicated that their oncology knowledge was extremely superficial and was included in a single lecture that was unrelated to oncology.

### Lack of a formal pathway to work in oncology

This subtheme discusses the challenges resulting from the lack of a structured career trajectory in the field of oncology nursing for recently qualified RN nurses. Usually, job advertisements for newly recruited nurses lack information regarding the specialization or particular department in which the nurse will be expected to work. Newly employed nurses in Saudi hospitals are required to participate in a 3-month rotation program to evaluate their nursing skills before being allocated to a specific department. Typically, nursing managers engage in discussions with nurses about their preferred specialty paths during their 3-month rotations. Many RN participants reported that during the rotation period, their specialty preferences were not taken into consideration:* “After I completed my 3-month oncology rotation, I went to the nursing office and told them that I would like to continue in the same department…But they refused, and the nursing director office assigned me to the operating theatre department”*. Conversely, other participants commented on the insufficiency of preparation before starting work in the department of oncology, particularly in skills relating to patient communication.

### Salary scale issue

Most participants highlighted financial considerations as the most important barrier to working in oncology. With the exception of psychiatric nurses, in Saudi government hospitals, nurses’ salaries adhere to a fixed salary scale system that is determined by the number of years of experience and not by the specialization. The majority of participants in both groups expressed dissatisfaction with a perceived unfair compensation for those employed in advanced speciality departments in comparison to other departments *“Saudi nurses in all nursing departments have the same salary rate; it’s unfair. I see those who work in outpatient clinics relaxed while the other departments work very hard”.* Other participants made a comparison between the financial compensation and the psychological stress of working in oncology “*take it from the perspective of why I work in a department that is so difficult when I can get the same salary if I work in a department that is not psychologically tiring for me…. The financial allowance: I expected it to make a difference*”.

## Workplace conditions that reduce job satisfaction

This main theme outlines the workplace environments that decrease job satisfaction in the specialty of oncology. Four subthemes were evident within this theme.

### Communication barriers

The communication barrier was the most recurrent topic discussed by participants due to the importance of effective communication skills when interacting with oncology patients and their families. All expatriate ORNs reported challenges in patient communication due to their low proficiency in Arabic and the patients’ inability to conduct conversations in English: *“I only know some medical terms in Arabic but not conversational Arabic”*. This issue became more complex when considering patients residing in rural regions “*Those from the city can understand English. They know that we can communicate half in English, half in Arabic, and something like that. But those from the rural areas really do not understand English, or sometimes their Arabic dialect is also different and very hard to understand. Then we really face difficulty in communicating with them, even in Arabic. And that’s why we do not ask them more questions”*. Consequently, most Saudi ORN participants discussed being used as translators in the department: “*Yes, I work as an interpreter…, which takes our time…It is a burden because it is not our job*”.

### Team-related issues

The ORNs identified issues that occurred when they had to fill in for perceived insufficiencies in the work of other members of the healthcare care, which led to an increased burden on them as nurses: *“The doctors didn’t explain; they were supposed to explain to the patient at the time of diagnosis before the treatment started. They should give the patients a sufficient explanation. But patients always asked me for an explanation and disease progression details, which increased our workload”*. Several ORN participants commented on the lack of spiritual services: *“We don’t have a spiritual team. Most of the time, we referred patients to the social worker or the psychological/psychiatric therapist”*.

### Work-related wellbeing

The majority of ORN participants emphasized that an inadequate nurse-to-patient ratio (NPR) had a detrimental impact on the quality of nursing care because of the restricted time that was available: *“shortages will increase the pressure on each nurse and possibly lead to poor patient service; the service will not be 100% bad but will reduce in quality”*. When the job went beyond their working hours, several ORN participants expressed their frustration “*even after we reach our homes, the doctors used to call us: What happened to that patient? We don’t accept that, but sometimes, we may be forced to do it; that is, you want to help and don’t have any other choice”*. Furthermore, many RN participants expressed their unwillingness to consider pursuing a career in oncology nursing. They cited the heavy workload and shortage of nurses in the department as the leading cause for their decision. Additionally, they highlighted the negative impact of this shortage on their ability to take annual leave while working in oncology: “*I did not like this specialty, because of the staff shortage, as well as difficulties to taking regular annual leave. Another negative aspect of this is that it is difficult to cover the shortage in oncology by nurses from other departments, it is not possible to bring staff from a normal ward to a critical area like the oncology, it is hard to cover. it also affects us in terms of vacations, for example, if your vacation time is starting soon, the hospital administration says no, why? Because of the lack of staff, we can't give you a vacation because there is no staff covering the department”.*

### Length of shift preference

Most hospitals in Saudi Arabia implement two different shift systems in their inpatient oncology departments, depending on local hospital policy. Some departments operate a 12-h shift schedule over four days per week, while others follow an 8-h shift schedule over five days per week. The study findings revealed that the type of shift system had a great impact on ORNs wellbeing. Most ORN participants indicated that they favored an 8-h shift schedule as opposed to a 12-h shift schedule: *“I would prefer 8-h shifts; yes. It is better than working 12 h because it’s very exhausting in oncology”*. This was also expressed by RNs who had been involved in oncology practice: *“to work a 12-h shift with oncology patients is very difficult, can’t tolerate that”*. Eight-hour shifts were favoured as they helped to decrease the stress of caring for terminally ill patients and their families “*Yes*, *for 8 h shift…Daily, you are faced with dying patients; daily, you are faced with a lot of problems from the family, especially the family that is in denial. It is very hard work”*. Additionally, some RN participants expressed that 8-h shifts were not typically available in the oncology specialty. They noted that if such shifts were an option, they would be more inclined to work in the field:*“If there were 8-h shifts, I would have chosen it from the beginning … I think even the number of Saudi nurses would increase”*.

## Discussion

The exploration of oncology nurses’ motivations and challenges revealed multifaceted and interrelated factors influencing their career choices and experiences. This discussion contextualizes our findings within the broader literature and provides insights into potential interventions to enhance job satisfaction and retention among oncology nurses in Saudi Arabia.

Oncology nurses in our study highlighted a strong sense of professional fulfillment and personal satisfaction as key motivators for their work. This intrinsic motivation aligns with findings from various studies indicating that the emotional rewards of patient care, such as seeing the positive impact on patients’ lives and forming meaningful connections, significantly contributes to job satisfaction [23–25]. Nurses described feeling a deep sense of accomplishment when they could provide compassionate care and support to patients during their cancer journey, which is consistent with the literature emphasizing the importance of emotional and psychological rewards in nursing [26, 27].

Despite the intrinsic rewards, the psychological demands of oncology nursing are considerable. Nurses reported high levels of emotional strain and burnout, driven by continuous exposure to patient suffering and death. The emotional labor associated with oncology nursing, coupled with inadequate psychological support, exacerbates these challenges [28, 29]. Similar findings have been documented in studies where high emotional demands and lack of support systems contribute to increased stress and burnout among nurses [30, 31]. The work and life quality of ORNs were negatively affected by emotional exhaustion, especially when patients suffered or died [32]. Interventions to provide regular psychological support and resilience training are crucial to mitigate these issues and sustain nurses’ mental health and job satisfaction.

Furthermore, the findings reveal a significant concern among female ORNs regarding their own future health outcomes due to their exposure to chemotherapy and radiation treatments, especially those planning pregnancy, due to fears of congenital deformities in their babies. These concerns are not unfounded, as no studies have yet been conducted in Saudi Arabia to assess the extent of harm to ORNs from handling these treatments. However, the National Institute for Occupational Safety and Health (NIOSH) in the USA has documented real risks of adverse health events associated with exposure to hazardous drugs, emphasizing the importance of personal protective equipment (PPE) [33]. Despite this, several studies indicate that many ORNs do not consistently use PPE [34–36]. Chronic exposure to hazardous drugs, even at low levels, has been linked to increased risks of adverse reproductive outcomes [37]. Therefore, oncology nurses who handle hazardous drugs must take PPE precautions in order to minimize both direct and indirect exposure to these drugs [38]. This potential harm and the associated fear may drive female ORNs, particularly those who are pregnant or planning to become pregnant, to consider leaving oncology nursing, highlighting a critical area for future research and intervention.

Our study identified several extrinsic factors that impact oncology nurses’ motivation and retention. Several barriers such as inadequate financial compensation, insufficient training opportunities, lack of formal career pathways, and inadequate staffing levels were prominent concerns. These findings are consistent in the literature, where organizational issues, including heavy workloads, insufficient nurse-to-patient ratios, and inadequate financial compensation, are frequently cited as contributing to job dissatisfaction and high turnover rates [4, 12, 39–42]. Addressing these organizational barriers through policy changes and resource allocation is essential to improve the work environment and retain skilled oncology nurses.

Furthermore**,** effective communication is crucial in oncology nursing, yet language barriers pose significant challenges, particularly for expatriate nurses as revealed in this study. The lack of proficiency in Arabic among expatriate nurses hinders effective patient-nurse interactions, leading to patient dissatisfaction and potential mis-communication [10, 43, 44]. Enhancing cultural competence through targeted language training and cultural sensitivity workshops can improve communication and patient care [8, 44–46]. Additionally, integrating professional interpreters into the healthcare team can support expatriate nurses in their interactions with patients and families [10].

The current study revealed that job satisfaction emerged as a key factor influencing nurses’ intention to remain in oncology nursing. Furthermore, the supportive management, opportunities for professional development, and recognition of their work were critical to nurse’s job satisfaction. Previous studies found that nurses who felt valued and supported by their organization were more likely to express a desire to continue working in oncology [42, 47]. Furthermore, one significant extrinsic factor hindering Saudi RNs from joining the oncology nursing workforce and retaining Saudi ORNs was the salary scale. Many Saudi nurses expressed dissatisfaction with the current salary scale system due to the absence of attractive financial rewards for working in an advanced nursing speciality (other than psychiatric nursing), stating that it did not reflect the complexity and demands of their work [40]. Competitive salaries are crucial to attracting and retaining skilled oncology nurses. Implementing a salary scale that recognizes the specialized skills and emotional labor involved in oncology nursing is essential. This adjustment can enhance job satisfaction and reduce turnover rates by ensuring that nurses feel valued and adequately compensated for their work [47–51]. In light of this, it is clear that addressing the issue of financial compensation is the first step in addressing the shortage of Saudi ORNs in Saudi hospitals.

Overall, poor work-related wellbeing among oncology nurses has profound implications for both individual practitioners and healthcare systems. Research consistently shows that inadequate wellbeing is linked to burnout, compassion fatigue, and moral distress, leading to decreased job satisfaction, higher turnover rates, and compromised patient care quality [52–54]. Oncology nurses often face emotionally demanding situations, such as complex ethical dilemmas and prolonged exposure to patient suffering, which intensify psychological stress and hinder their ability to provide compassionate care​ [11, 55]. Addressing these challenges requires targeted interventions, including resilience training, psychological support, and organizational changes to optimize workloads and job satisfaction​ [52, 56]. Furthermore, cultivating a supportive organizational culture that prioritizes nurses’ mental health is critical for mitigating these stressors and ensuring workforce sustainability. Advancing research in this area is essential to develop effective strategies that empower oncology nurses to thrive while delivering high-quality, patient-centered care.

## Strengths and limitations

This study provides in-depth insights into the motivations and challenges faced by oncology nurses in Saudi Arabia, offering valuable qualitative data from semi-structured interviews. Its focus on the specific cultural and organizational context of Saudi Arabia enhances the relevance of the findings for local healthcare policies and practices. The inclusion of both expatriate and local nurses adds diversity to the sample, providing a comprehensive view of different perspectives.

As the first study of its kind in Saudi Arabia, it sets a foundation for future research in this area. However, the study’s limitations include being conducted in a single setting, which may limit the transferability of the findings. Additionally, the qualitative nature of the study means that findings are based on subjective experiences and interpretations, which may not capture the full range of perspectives. Despite these limitations, the study significantly contributes to understanding oncology nurses’ experiences and can inform targeted interventions to improve job satisfaction and retention.

## Implications for policy and practice

The insights from this study have significant implications for healthcare policy and practice in Saudi Arabia. Addressing both intrinsic and extrinsic factors that influence oncology nurses’ motivation and retention requires a multifaceted approach. Policymakers and healthcare leaders should consider the following recommendations:

1. Enhance Training and Education: Support the oncology curriculum at the undergraduate level. Additionally, provide essential training opportunities in this field for undergraduate students to equip them with the necessary skills and knowledge.
2. Improve Psychological Support: Implement regular psychological support and resilience training programs to help nurses cope with the emotional demands of oncology nursing.
3. Address Organizational Barriers: Ensure fair workload distribution, adequate staffing levels, and competitive salaries to improve job satisfaction and reduce turnover rates.
4. Promote Cultural Competence: Provide language training and cultural sensitivity workshops to enhance communication between expatriate nurses and Saudi patients.
5. Foster a Supportive Work Environment: Create a culture of appreciation and support, offering opportunities for professional development and recognizing the contributions of oncology nurses.

## Conclusion

This study provides valuable insights into the motivations and challenges faced by oncology nursing speciality in Saudi Arabia. By addressing the identified factors through targeted interventions and supportive policies, healthcare leaders can enhance the recruitment and retention of oncology nurses in Saudi Arabia, ultimately improving the quality of cancer care. Future research should aim to identify and evaluate specific interventions and policies that can be implemented across different healthcare environments to support and sustain oncology nurses in their essential work.

## Data Availability

The datasets used and analysed during the current study are available from the corresponding author on reasonable request.

## Abbreviations

RN: Registered Nurse
ORN: Oncology Registered Nurse
KSUMC: King Saud University Medical City
